# Kidney transplantation beyond immunosuppression: shifting the focus from graft survival to patient health

**DOI:** 10.3389/fimmu.2026.1738675

**Published:** 2026-01-28

**Authors:** Gabriella Moroni, Francesco Reggiani, Claudio Ponticelli

**Affiliations:** 1 Nephrology and Dialysis Unit, IRCCS Humanitas Research Hospital, Milan, Italy; 2 Department of Biomedical Sciences, Humanitas University, Milan, Italy; 3 Independent investigator, Milan, Italy

**Keywords:** cancer, cardiovascular diseases, diet, healthy lifestlye, immunosuppressants, infections, kidney transplantation, smoking

## Abstract

Kidney transplantation has traditionally focused on maximizing graft survival, with immunosuppressive therapy as the cornerstone of care. However, long-term patient health requires a broader perspective that addresses the prevention and management of post-transplant complications. This review explores strategies extending beyond immunosuppression, emphasizing the joint responsibility of patients and clinicians in achieving optimal outcomes. Patients play a pivotal role through adherence to lifestyle measures, including regular physical activity, balanced nutrition, meticulous hygiene, infection prevention, and sun protection to reduce skin cancer risk, as well as strict compliance with prescribed therapies. These self-care practices can significantly lower the incidence of infections, cardiovascular disease, and malignancies. Clinicians, in turn, must adopt a proactive, comprehensive approach to surveillance and early intervention. This includes timely recognition and management of cardiovascular complications, vigilant cancer screening, and infection control tailored to individual risk profiles. By integrating patient education, lifestyle optimization, and vigilant clinical oversight, kidney transplant care can shift from a narrow focus on graft survival toward a holistic model prioritizing overall patient well-being and longevity. Such an approach recognizes that the success of transplantation is measured not only in years of graft function, but in the quality and health of the patient’s life.

## Introduction

The modern era of kidney transplantation (KT) began in 1954 with the first successful kidney transplant between identical twins. Subsequently, kidney allografts from living and deceased donors were performed using immunosuppressive regimens based on steroids and azathioprine, although acute rejection remained a major cause of graft failure. A major advance occurred in the 1970s with the introduction of cyclosporine, which significantly reduced rejection rates and improved long-term graft survival. Further progress was achieved with the availability of tacrolimus, a more potent calcineurin inhibitor, and mycophenolate mofetil, a more effective purine synthesis inhibitor. Currently, triple immunosuppressive therapy with tacrolimus, mycophenolate mofetil, and steroids has substantially reduced the risk of acute rejection and improved both graft and patient survival (1). Yet, the management of kidney transplant recipients (KTRs) remains one of the most challenging areas in clinical medicine. Effective clinical practice demands not only comprehensive knowledge of immunology and pharmacology, but also multidisciplinary expertise in cardiology, infectious diseases, oncology, dermatology, and renal pathology, supported by close collaboration with specialized teams. Despite these complexities, KT outcomes have progressively improved over recent decades. Nevertheless, KTRs continue to experience a wide spectrum of disabling and potentially life-threatening complications. Consequently, contemporary management of KTRs should extend beyond immunosuppressive therapy to include the prevention and early detection of complications that may negatively impact life expectancy and quality of life. Optimal care therefore requires heightened awareness and coordinated efforts from both transplant recipients and healthcare providers to minimize the burden of the most common post-transplant complications.

**Figure 1:**
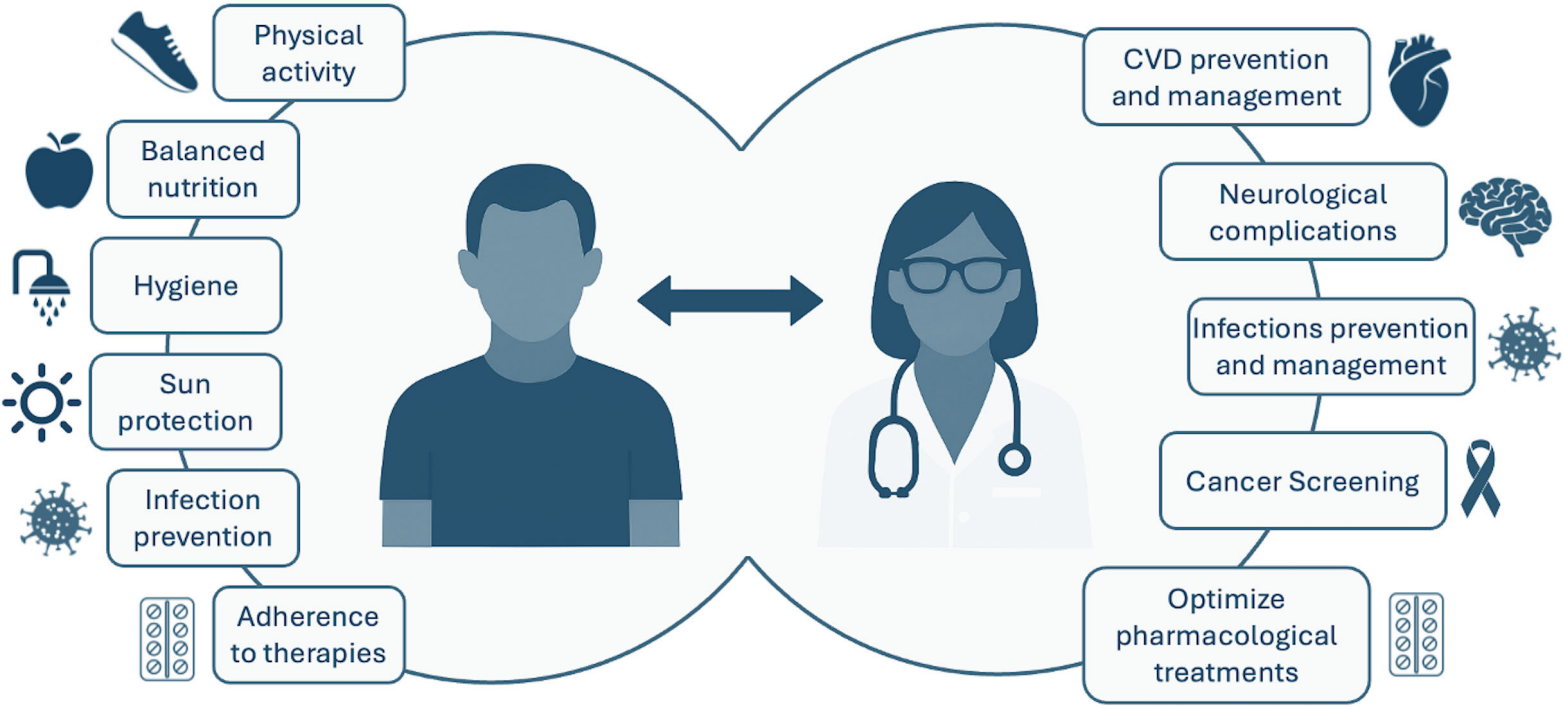
Post-transplant actions to improve long-term health and well-being. The infographic highlights key areas of action-on the patient’s side, lifestyle measures and treatment adherence; on the physician’s side, prevention, monitoring, and proactive management of complications. CVD, cardiovascular diseases.

KTRs should actively participate in their long-term care to preserve graft function and overall health. They should maintain a healthy style of life, collaborate with healthcare providers, and pay the utmost attention and care to prevent the most common complications (**Figure 1**).

## Materials and methods

We performed a structured literature search using PubMed and Embase to identify relevant publications. Inclusion criteria comprised peer-reviewed articles published within the last 10 years, with a limited number of exceptions, and focused on post-kidney transplant complications and long-term patient outcomes. Exclusion criteria included non-peer-reviewed sources and case reports. Each selected article was assessed for relevance, methodological quality, and potential bias. Strength of evidence was evaluated using a modified hierarchy prioritizing systematic reviews, meta-analyses, and randomized controlled trials (RCTs), followed by observational studies. Risk of bias was appraised using standard criteria, including study design, sample size, outcome measures, and potential conflicts of interest. Evidence was synthesized qualitatively, with emphasis on findings from higher-quality studies.

## What the patient should do

Although many health maintenance recommendations for KTRs may appear similar to those provided to the general population, their clinical relevance, intensity, and consequences differ substantially in the transplant setting. In KTRs, chronic immunosuppression, persistent low-grade inflammation, altered immune surveillance, and drug–metabolism interactions profoundly modify the risk–benefit balance of lifestyle behaviors. Measures that are preventive in healthy adults become critical therapeutic strategies in transplant recipients, as failure to adhere may translate into accelerated cardiovascular disease, infection, malignancy, and graft loss rather than long-term health alone. Moreover, certain recommendations require specific adaptations, stricter monitoring, or carry unique implications in KTRs, such as the infection risk related to hygiene practices, dietary interactions with immunosuppressive drugs, or the markedly increased susceptibility to skin cancer. The following sections therefore highlight not only general lifestyle principles, but also their transplant-specific implications.

### Following a healthy style of life

A healthy lifestyle is critical to prevent complications, including graft dysfunction, cardiovascular disease (CVD), infections, and malignancies.

#### Smoking cessation

Smoking is a major risk factor for both patient and graft survival in KTRs (2). Smoking increases inflammatory markers and stimulates the expression of adhesion molecules such as integrins, cadherins, selectins, and cytokines across various tissues such as Interleukin-1 beta (IL-1β), Interleukin-6 (IL-6), Interleukin-8 (IL-8), and tumor necrosis factors (TNFs); and enhance the expression of chemotactic cytokines, such as recombinant human C-X-C Motif Chemokine Ligand 9 (CXCL9/MIG), thymus and activation-regulated chemokine (TARC), and IFN-inducible T cell α chemoattractant (ITA) (3). DNA damage response caused by smoking and immunological response are linked by immune effectors such as the cyclic GMP-AMP synthase (cGAS)–Stimulator of Interferon Genes (STING) pathway. These effectors act as sensors of DNA damage-caused immune response along with the activation of innate immune pathways, amplifies the expression of pro-inflammatory cytokines (4). Smoke-induced oxidative stress and inflammation also affect platelet adhesion, aggregation, and coagulation by upregulating adhesion molecules (5). Finally, smoking alters the balance between the coagulation cascade and fibrinolysis, favouring thrombus formation. Overall, exposure to smoke significantly impairs endothelial function while promoting inflammation and thrombosis, thus contributing to the onset and progression of atherosclerosis and related atherothrombotic cardiovascular diseases (6). Moreover, graft microvascular atherothrombosis represents a major concern, as the inflammatory state itself is a recognized risk factor for both cardiovascular disease and graft fibrosis in kidney transplantation failure (7). Smokers face a 25-fold higher risk of developing lung cancer compared to those who have never smoked (8). Additionally, patients who continue to smoke after transplantation have an increased risk of graft failure although the pathogenesis of smoking-related kidney damage remains largely unknown (9). Multiple interconnected pathophysiological pathways may underlie this association, including smoking-induced endothelial dysfunction, increased oxidative stress, and activation of inflammatory cascades, which can impair renal microcirculation and promote fibrotic changes within the graft (10). In addition, smoking may adversely interact with immunosuppressive therapies by altering drug metabolism and increasing susceptibility to infections and cardiovascular complications, thereby indirectly compromising graft outcomes (10). Smoking-related kidney damage is frequently marked by interstitial fibrosis. A history of smoking before KT can still significantly contribute to allograft loss (11).

#### Physical activity

Among the many modifiable factors that can increase the risk of morbidity and mortality in KTRs, physical inactivity is often neglected. Physical exercise helps prevent cancer (12), CVD (13), diabetes (14), and osteoporosis (15). In adults with chronic kidney disease (CKD), physical activity can improve obesity (16) and has beneficial effects on physical fitness, cardiovascular health, quality of life, and nutritional parameters (17). Low physical activity at the time of kidney transplantation is a strong predictor of all-cause mortality, particularly among older recipients, smokers, and individuals with diabetes. In a multivariable Cox regression analysis, higher levels of physical activity were strongly associated with improved survival (13). Nevertheless, most KTRs—especially elderly individuals and children—tend to lead sedentary lifestyles. Among older adults, those referred for KT generally demonstrate lower physical performance than peers with other chronic diseases and are at heightened risk of disability during the waiting period. Most children with CKD also exhibit low exercise capacity and physical inactivity (18). Even after successful transplantation, many patients continue to see themselves as disabled and remain inactive, with only one-fourth engaging in physical exercise (19). Progressive physical activity is recommended for these patients. A systematic review showed that regular physical activity after KT is associated with improved quality of life and aerobic fitness and with reduced body fat (20). A systematic review of 16 RCTs found that exercise training may provide several benefits for KTRs, including improvements in cardiorespiratory function, exercise capacity, strength, HDL cholesterol levels, maximum heart rate, and quality of life (21). Another systematic review of 11 RCTs reported that exercise interventions in KTRs improve arterial stiffness, exercise tolerance, and quality of life, even if they do not consistently improve other cardiovascular risk factors such as blood pressure, lipid profile, glycemic control, kidney function, body weight, or body mass index (22). However, patients who engage in physical activity should inform their providers. Creatinine is a by-product of muscle metabolism, and intense exercise may lead to an increase in serum creatinine that could alarm the transplant clinician (23).

#### Adopting a proper dietary regimen

The dietary approach in kidney transplant recipients is summarized in **Table 1** (24). As a general guideline, the diet should be normocaloric or hypocaloric, low in fats, low in sodium and designed to prevent osteoporosis. KTRs should aim to maintain an ideal body weight for their age and avoid both overweight and underweight conditions. Many KTRs gain weight after transplantation, and some become obese — a condition that increases the risk of acute rejection and graft loss (26, 27), probably through an association with early postoperative complications as well as with other medical conditions such as type 2 diabetes mellitus, cardiovascular disease, and chronic respiratory disorders which also predispose to metabolic syndrome (28). For transplant recipients, a body mass index of 40 kg/m² is the most common threshold for referral to medical or surgical weight-loss interventions. Most programs (73.4%) offer weight-management services within their institution, while 19.4% have programs integrated directly into their transplant services. One of the most frequently cited reasons for not referring patients for weight management was a preference for management by primary care providers or general nephrologists, particularly in the pre-transplant setting (16). Moreover, many KTRs exhibit arterial hypertension and/or dyslipidemia, and approximately 15–20% of patients with no previous history of diabetes develop diabetes within six months after transplantation, while 20–30% develop diabetes during long-term follow-up (29–31). Excess dietary sodium is a major contributor to hypertension. Volume expansion, impaired kidney function and sodium imbalance, impaired responses of the renin–angiotensin–aldosterone system (RAAS), central stimulation of sympathetic nervous system activity, and potentially inflammatory processes may explain salt-dependent hypertension (32). The Kidney Disease Outcomes Quality Initiative (KDOQI) Clinical Practice Guidelines for Nutrition in Chronic Kidney Disease recommend that kidney transplant recipients limit sodium intake to less than 100 mmol/day (approximately <2.3 g/day). It has been shown that reducing salt intake is associated with improved blood pressure control, better graft function, and more favorable metabolic parameters (25, 33). A retrospective analysis of 308 KTRs demonstrated a positive correlation between dietary salt intake and the slope of eGFR. A low-salt diet was associated with a more rapid annual decline in eGFR, with an adjusted odds ratio of 2.40 (95% confidence interval, 1.18–4.90). These findings suggest that current recommendations for sodium intake in kidney transplant recipients may warrant reassessment (34). Patients should avoid foods with hidden high sodium content, such as cured meats and most processed or packaged products (35).

**Table 1:** Dietary recommendations in kidney transplant recipients (24, 25).

Nutritional aspect	Recommendation
Overall diet composition	
Proteins (≈20%)	Fish, lean meat, skinless poultry
Fats (≈30%)	Olive oil, well-cooked eggs, fish oil, dairy products
Carbohydrates (≈50%)	Cereals, vegetables, fruits, whole grains
Dietary precautions	
High risk food list	Meat, fish and poultry if raw or undercooked: • Meat • Poultry • Fish • Crustaceans Dairy products: • Unpasteurized milk • Uncooked or undercooked eggs Fruits and vegetables: • Unwashed raw fruits and vegetables • Unpasteurized juices or ciders • Sprouts (like bean sprouts)
Limit salt consuption	Salt limited to 2,300 mg (100 mmol) per day for adults, avoiding: • Table salt • Cured meats (e.g. bacon, ham or sausages) • Lunch meats (e.g. salami or hot dogs) • Pre-packaged frozen dinners • Soy sauce • Canned soups and pasta sauce • Pickled foods (e.g. olives or pickles) • Snack foods (e.g. salted chips, nuts, pretzels, and popcorn)
Reduce weight gain	Eating foods high in fiber Choosing lean meats Having nonfat dairy products Drinking mostly water, with some unsweetened tea, coffee or milk Avoiding soft drinks and sodas Avoiding fried foods, saturated fats, trans fats Limit alcohol consumption
Avoid food that interact with drugs	Grapefruit and grapefruit juice Pomegranate Bitter orange

### Hygiene and infection prevention

Handwashing remains the easiest and most effective method of preventing infections. However, despite the simplicity of the technique, the adherence rate among patients and health professionals remains suboptimal (36, 37). The genital and anal areas should be cleaned thoroughly. Frequent use of nystatin swish and swallow is recommended during the early post-transplant period to prevent oral and esophageal fungal infections. Showers should be taken every 2–3 days or after any activity that causes sweating (38). Regular tooth brushing and flossing can help prevent periodontitis, a multifactorial chronic inflammatory disease, which may increase the risk of CVD (39). Patients should ventilate their rooms regularly, wear clean clothes, and change them frequently. They should also avoid crowded places and passive smoking (38).

### Maximizing sun protection

In KTRs, the incidence of skin cancer exceeds that of the general population, largely due to long-term immunosuppressive therapy, with risk further modulated by cumulative ultraviolet (UV) exposure (40). Accumulation of UV radiation generates stress to cells and organisms by impeding DNA replication and transcription. Moreover, UV radiation can broadly induce ROS which usually lead to cell death, such as apoptosis and necrosis (41). Basal cell carcinomas and squamous cell carcinomas are the most frequent types of skin cancer. Older age at transplant was significantly associated with a higher incidence of non-melanoma skin cancer (42). Preventive skin protection measures are the same as those recommended for the general population; however, in KTRs greater awareness and adherence are required due to their increased risk of skin cancer (43). Therefore, minimizing sun exposure and avoiding tanning beds are essential for preventing skin cancer in transplant recipients. Patients should wear long-sleeved shirts and pants, along with a hat and sunglasses, when outdoors. They should use regularly broad-spectrum sunscreen, which protects against both UVA and UVB radiation. Frequent self-examination of the skin is also recommended. Whenever possible, patients should take a photo of any suspicious skin lesion, even with a smartphone, as this can be helpful for early detection and monitoring of skin tumors (44).

### Ensuring strict adherence to prescriptions

In KTRs, adherence to prescribed medications is often compromised by the complexity of treatment regimens. Transplant medications can cause a wide range of side effects, including cosmetic changes, gastrointestinal disturbances, neurological complications, hyperglycemia, and hypertension. Depression is common among transplant candidates and recipients, with a reported prevalence of up to 85.8% (45), and significantly impairs both medication adherence and clinical outcomes. Non-adherence is particularly prevalent among adolescents. In some cases, patients intentionally do not follow the prescribed treatment as a way of challenging their providers, in order to see whether they notice the non-adherence (46).

A systematic review analyzed rates of non-adherence fluctuating from 1.6 to 96% in 37 studies. Youth (≤50 years old), male, low social support, unemployment, low education, ≥3 months post graft, living donor, comorbidities, more than 5 drugs, negative beliefs, negative behavior, depression and anxiety were the factors significantly related to non-adherence (47). Among older patients, unintentional non-adherence often results from forgetfulness—especially as medication regimens become more complex. Changes in drug type or dosage may contribute to confusion.

### Alarm signs

Beyond preventive measures and lifestyle recommendations aimed at preserving graft function and improving quality of life, patients should be educated to promptly recognize and report clinical warning signs suggestive of post-transplant complications. Early identification of these symptoms is essential, as delays in evaluation and treatment may result in rapid clinical deterioration, increased morbidity, and adverse graft outcomes.

Patients should immediately contact their caregiver in the event of fever >38 °C, tenderness or pain over the transplanted kidney, severe headache, dyspnea, reduced urine output, edema, hematuria, or respiratory or gastrointestinal symptoms (48). The occurrence of one or more of these manifestations warrants urgent clinical assessment and often hospital admission, as they may represent the first clinical expression of infection, acute rejection, drug-related to5xicity, or other transplant-related complications.

## Complications the medical team should aim to prevent

### Graft function

Serum creatinine is the most widely used marker for monitoring kidney transplant function. However, creatinine is a metabolite of creatine and therefore a by-product of muscle metabolism. Its levels can be influenced by several non-renal factors. Increased creatinine production may result from a high intake of meat or protein (particularly shortly before testing), as well as from intense physical exercise or muscle injury (e.g., rhabdomyolysis., trauma, seizures) (49, 50). Additional causes of spurious serum creatinine elevation include assay-related interferences. With the classic colorimetric method, high glucose levels, bilirubin, certain cephalosporins, and barbiturates can lead to falsely elevated results. Although more specific, enzymatic assays may still be affected by substances such as vitamin C or dopamine infusion (49, 50). Despite these limitations, serum creatinine remains a reliable parameter for raising suspicion of acute rejection. Nevertheless, an increase in creatinine exceeding 0.3 mg/dl above baseline may represent a spectrum of non-immunological injuries rather than acute rejection itself. Most transplant clinicians rely on therapeutic drug monitoring (TDM) to guide tacrolimus dosing. Tacrolimus has a narrow therapeutic window and exhibits high interindividual pharmacokinetic variability (51). Conventional TDM measures whole-blood concentrations, which largely represent inactive fractions, whereas the immunosuppressive activity of tacrolimus is exerted within T cells. Measuring intracellular tacrolimus concentrations remains technically challenging, and available studies have reported conflicting findings (52). Inhibitors of CYP3A4 (such as azoles, macrolides, and grapefruit) can elevate tacrolimus levels by slowing its metabolism, thereby increasing the risk of toxicity. Conversely, inducers of CYP3A4 (including rifampin, phenytoin, carbamazepine, phenobarbital, and hypericum perforatum) can reduce intracellular tacrolimus levels, increasing the risk of graft rejection (53).

P-glycoprotein is a membrane transporter protein that pumps various substances, including drugs and toxins, out of cells. Strong P-glycoprotein inhibitors (such as verapamil, quinidine, ketoconazole, itraconazole, ritonavir, and amiodarone) block the efflux of tacrolimus from intestinal cells, hepatocytes, and renal tubular cells. As a result, reduced efflux of tacrolimus leads to higher systemic and intracellular exposure (54). However, elevated intracellular concentrations have been associated with an increased risk of nephrotoxicity and neurotoxicity. Conversely, P-glycoprotein inducers (such as rifampin, hypericum perforatum, carbamazepine, phenytoin, and phenobarbital) may reduce intracellular tacrolimus levels even when blood concentrations appear adequate (55), thus increasing the risk of underexposure and graft rejection. Recognition of these modulatory mechanisms is therefore crucial for safe and effective calcineurin inhibitor therapy in transplant recipients. Transplant physicians who continue to rely on blood-level measurements should interpret them with a thorough understanding of pharmacological agents that may interfere with therapeutic drug monitoring. Maintaining an up-to-date and readily accessible list of drugs known to affect CYP3A4 and P-glycoprotein function is strongly recommended to minimize the risk of potentially serious dosing errors.

Apart from graft biopsy findings, biomarkers for detecting subclinical or acute rejection include urinary CXCL9 and 10 (56). Nevertheless, these biomarkers can reflect various forms of acute kidney injury, not exclusively clinical or subclinical rejection. For this reason, they should always be interpreted in conjunction with clinical data and, when appropriate, biopsy findings to ensure diagnostic accuracy. Of particular interest is donor-derived cell-free DNA (dd-cf DNA) for non-invasive rejection monitoring (57). A multicentre study involving 1,134 patients demonstrated that dd-cfDNA (%) was significantly correlated with allograft rejection, including antibody-mediated rejection, T cell–mediated rejection, and mixed rejection. Multivariable analysis further showed that circulating dd-cfDNA was independently associated with allograft rejection, irrespective of standard patient monitoring parameters. Moreover, dd-cfDNA exhibited a high predictive value for detecting subclinical rejection in clinically stable patients and may contribute to reducing unnecessary biopsies (58).

Despite its superior specificity for detecting rejection compared with other biomarkers, it should be noted that dd-cfDNA may also reflect other causes of AKI, including drug toxicity and viral infection.

### Cardiovascular diseases

CVD is a leading cause of morbidity and mortality following KT (59, 60). Early post-transplant cardiovascular complications are largely attributable to the high burden of pre-existing comorbidities, including hypertension, glucose intolerance, dyslipidemia, and ischemic heart disease. Immunosuppressive therapy may further potentiate these risks by exacerbating hypertension, dyslipidemia, and diabetes. In addition, some KTRs continue to smoke, which further increases their cardiovascular risk (61). Other contributing factors—including vascular calcification, left ventricular hypertrophy, and inflammation—are often present before transplantation and may worsen afterward (61–64).

Hypertension is the strongest risk factor for CVD. It is highly prevalent following KT, affecting approximately 50% to 80% of KTRs (62, 63). Clinically, it is often described as a persistent or newly developed elevation in blood pressure following successful KT. In addition to negatively impacting renal allograft outcomes, post-transplant hypertension is strongly associated with an increased risk of CVD (65). A Collaborative Transplant Study demonstrated that higher systolic blood pressure is independently associated with a greater risk of CVD and all-cause mortality in KTRs (64). Transplant renal artery stenosis is a relatively common vascular complication that can also lead to allograft dysfunction. Its pathogenesis involves mechanical vascular injury during surgical anastomosis, as well as immune-mediated endothelial damage (66).

The target blood pressure in this population should be below 130/80 mmHg, irrespective of other risk factors. A study designed to assess whether achieving the blood pressure targets recommended by the American College of Cardiology/American Heart Association (ACC/AHA) hypertension guidelines provides additional benefit did not demonstrate improved graft survival or patient mortality with blood pressure reduction below 120/80 mmHg during the first year post-transplant, showing no difference between patients with normal blood pressure (<120/< 80 mmHg) and those with elevated blood pressure (120–129/< 80 mmHg). In contrast, hypertension stages 1 (130–139/80–89 mm Hg) and 2 (≥ 140/≥ 90 mm Hg) were associated with an 11% and 55% increased risk of death-censored graft failure, respectively (67). Medications with proven cardiovascular benefits in the general population are also commonly used in KTRs, including calcium channel blockers, beta-blockers, diuretics, and RAAS inhibitor. Bilateral nephrectomy of the native kidneys may be considered in cases of resistant hypertension (68).

New-onset diabetes after transplantation (NODAT) typically develops within 2–3 months post-transplant but can also occur several years later. Risk factors include older age, Hispanic or African American ethnicity (69), hepatitis C virus infection, and the presence of metabolic syndrome. Immunosuppressive agents—particularly glucocorticoids, tacrolimus, cyclosporine, and mTOR inhibitors—are also significant contributors to NODAT (70). CVD undoubtedly represents the most relevant and dreadful complication of NODAT (71). Preventive measures include dietary modification, regular physical activity, weight loss in obese individuals, smoking cessation, and judicious reduction of glucocorticoid and calcineurin inhibitor exposure when clinically feasible. Management of NODAT involves comprehensive control of glycemia, blood pressure, and lipid levels. In recent years, the use of glucagon-like peptide-1 receptor agonists (72), sodium-glucose cotransporter 2 inhibitors (73), and finerenone (74) have demonstrated particular benefit in KTRs with NODAT, attributable to their potential cardiovascular and renoprotective effects (75).

Lipid disorders are common among KTRs and contribute significantly to cardiovascular morbidity and mortality (76, 77). Several factors predispose individuals to dyslipidemia including advanced age, smoking, diabetes, obesity, excessive intake of calories, saturated fats, alcohol, and sugar-sweetened foods or beverages, along with physical inactivity, hypothyroidism, proteinuria, renal insufficiency, use of beta-blockers or diuretics, and genetic predisposition. Immunosuppressive therapy also plays a key role in the development of dyslipidemia. Tacrolimus has a minimal effect on post-transplant hypercholesterolemia, although hypertriglyceridemia may occur due to reduced lipoprotein lipase activity (78). In contrast, cyclosporine downregulates LDL receptor expression through increased levels of proprotein convertase subtilisin/kexin type 9 (PCSK9), which promotes LDL receptor degradation. Cyclosporine also inhibits cholesterol 7-alpha-hydroxylase, thereby reducing intestinal cholesterol excretion (79), mTOR inhibitors are frequently associated with hypercholesterolemia, although the mechanisms remain incompletely understood. A recent meta-analysis demonstrated that both lipoprotein(a) and LDL cholesterol (LDL-C) are independent and additive contributors to atherogenic risk, and that LDL-C lowering alone does not fully mitigate the risk associated with elevated lipoprotein(a) (80).

Until recently, the primary goal of lipid-lowering therapy was to reduce LDL-C levels, with targets of ≤70 mg/dL in patients at high cardiovascular risk (81). Standard treatment was based on statins and ezetimibe, while proprotein convertase subtilisin/kexin type 9 (PCSK9) inhibitors and bempedoic acid were reserved for more difficult-to-treat cases (**Table 2**) (81). In KTRs, dyslipidaemia is highly prevalent and is driven both by pre-existing chronic kidney disease and by the metabolic effects of immunosuppressive therapies, which may increase total cholesterol, very-low-density lipoprotein (VLDL), triglycerides, and alter low-density lipoprotein (LDL) particle size and density. As a consequence, KTRs are at increased risk of atherosclerotic cardiovascular disease and transplant arterial vasculopathy (82).

**Table 2:** A possible algorithm to manage post-transplant LDL cholesterol and reduce cardiovascular risk.

Intervention steps	Action	Objective/Efficacy	Side effects
Diet	Vegetables, fruits, whole grains, legumes, healthy protein sources, olive oils	Maintain the ideal body weight	//
Step 1 Statins	The first drug to be used. Statins inhibit the 3hydroxymethylglutaryl-Coenzime A reductase, and convert it into mevalonate, the precursor of Cholesterol	Reductions in total- and LDL-Cholesterol of 20 to 55% based on different potencies and dosages	Myopathies, increases in transaminases, and rare new onset diabetes
Step2 Ezetimibe	If LDL cholesterol goals are not met with maximally tolerated statin therapy, then ezetimibe is often considered as the next addition. It inhibits the intestinal reabsorption of cholesterol	Coadministration with a statin in severe hypercholesterolemia	Rare cases of abdominal pain and/or diarrhea.
Step 3 PCSK9 inhibitors	If LDL cholesterol remains above target despite statin + ezetimibe, PCSK9 inhibitors (evolocumab, alirocumab) may be considered, especially in high-risk patients. They prevent degradation of LDL receptor.	Reduction of 52 to 70% of LDL-Cholesterol	Flu-like symptoms and muscle aches.
Step 4 Bempedoic acid	Blocks the ATP citrate lyase that activates HMG-CoA reductase.	Allows LDL-Cholesterol lowering up to 63%.	Flu-like symptoms. Reversible increases of creatinine and uric acid.

ATP, adenosine triphosphate; HMG-CoA, 3-hydroxy-3-methylglutaryl-coenzyme A; LDL-C, low-density lipoprotein cholesterol; PCSK9, Proprotein Convertase Subtilisin/Kexin type 9 (81).

The management of dyslipidaemia in transplant recipients generally follows recommendations for patients at high or very high cardiovascular risk, although particular attention must be paid to drug–drug interactions and immunosuppression-related adverse effects. Statins remain the cornerstone of lipid-lowering therapy due to their inhibition of HMG-CoA reductase, leading to reduced hepatic cholesterol synthesis and upregulation of LDL receptors. However, evidence for their clinical benefit in renal transplant patients is largely extrapolated from studies in patients with moderate reductions in glomerular filtration rate, as randomized outcome trials in KTRs are limited. The main adverse effects of statins include myopathy, elevations in liver enzymes, and, rarely, rhabdomyolysis, with the risk markedly increased by interactions with calcineurin inhibitors, particularly ciclosporin, which raises systemic statin exposure through CYP3A4 inhibition (83). Statins with lower interaction potential, such as fluvastatin, pravastatin, and rosuvastatin, are therefore preferred in this population (81).

Ezetimibe inhibits intestinal cholesterol absorption by targeting the Niemann–Pick C1-like 1 (NPC1L1) transporter and may be used as monotherapy in statin-intolerant patients or in combination with the maximally tolerated statin dose (81). Although no cardiovascular outcome data are available in transplant recipients, ezetimibe is generally considered a second-line option. Its use requires caution in patients receiving ciclosporin, as this drug can increase ezetimibe plasma levels by up to 12-fold (81). Gastrointestinal intolerance and mild elevations in liver enzymes represent the most common adverse effects.

PCSK9 inhibitors, which increase hepatic LDL receptor recycling by preventing PCSK9-mediated receptor degradation, may be considered in selected KTRs with severe hypercholesterolaemia or statin intolerance. Available evidence suggests a potent LDL-C–lowering effect with a favourable safety profile and minimal drug–drug interactions (81); however, data in transplant populations remain limited. The most frequent side effects include injection-site reactions and influenza-like symptoms. Reducing lipoprotein(a) levels in KTRs remains challenging, as concentrations are largely genetically determined. PCSK9 inhibitors may lower lipoprotein(a) plasma levels by approximately 20–30%, whereas the most effective strategy to date remains regular lipoprotein apheresis (84).

Bempedoic acid, an adenosine triphosphate–citrate lyase inhibitor that reduces hepatic cholesterol synthesis upstream of HMG-CoA reductase, represents an additional option in difficult-to-treat patients. Its activation is liver-specific, potentially limiting muscle-related side effects (85); however, data in transplant recipients are scarce. Reported adverse effects include hyperuricaemia, gout, and tendon rupture.

In summary, the prevention of CVD in KTRs is of paramount importance to reduce early morbidity and mortality. However, this task is complex. Transplant clinicians should regularly monitor not only blood pressure, glycated hemoglobin (HbA1c), and lipid disorders, but also assess whether new medications may interact with immunosuppressive therapy. In kidney transplant recipients, evidence to guide the optimal frequency of metabolic monitoring is limited and largely based on expert consensus. According to KDIGO/KDOQI recommendations, a complete lipid profile should be measured 2–3 months after transplantation, 2–3 months after any change in therapy or clinical condition affecting lipid metabolism, and at least annually thereafter. Glycaemic monitoring is recommended more frequently in the early post-transplant period: HbA1c and/or fasting glucose should be assessed weekly during the first 4 weeks, every 3 months during the first year, annually thereafter, and after initiation or dose escalation of calcineurin inhibitors, mTOR inhibitors, or corticosteroids (44). Close collaboration with relevant specialists is essential.

### Infections

Infections represent the most frequent cause of morbidity and the second leading cause of death in KTRs. Several precautions are recommended to reduce the risk of infection (see *Hygiene and infection prevention* section). Vaccination remains an underutilized tool both before and after transplantation. Overall, inactivated or conjugated non-live vaccines appear to be immunogenic, protective, and safe. Live vaccines, on the other hand, should be avoided in immunosuppressed individuals. A major issue with pretransplant vaccination is represented by a poor response to the vaccine due to the impaired immune response in patients with severe CKD (86). However, all approved inactivated vaccines are recommended according to standard schedules. Hepatitis B vaccination is strongly advised, ideally before transplantation, with measurement of anti-HBs titers 6–12 weeks after completing the series and annually thereafter; revaccination is suggested if titers fall below protective levels. Routine immunizations, except for annual inactivated influenza vaccination, are generally deferred for the first 3–6 months post-transplant and resumed once patients are on stable maintenance immunosuppression. Additional vaccines, such as pneumococcal, meningococcal, rabies, tick-borne encephalitis, Japanese encephalitis (inactivated), and typhoid vaccines, may be considered based on age, exposure risk, travel, or epidemiological factors (44, 87). Cytomegalovirus (CMV) disease is common in KTRs. Diagnosis can be confirmed through serology, qualitative and quantitative polymerase chain reaction, pp65 antigenemia, culture, and/or histopathology (88). Viral load and CMV pp65 antigenemia can also be used to monitor CMV replication. Prevention of CMV disease and its consequences is based on either a prophylactic or a preemptive strategy. Prophylaxis involves early and prolonged administration of ganciclovir or valganciclovir, whereas the preemptive approach relies on CMV antigenemia or DNA surveillance (88). A systematic review and meta-analysis concluded that prophylaxis is superior to the preemptive approach in preventing CMV infection during the first year after KT. The risk of acute rejection is also lower with the prophylactic strategy, although no significant differences have been observed in graft loss or mortality between the two approaches (89). Long-term prophylaxis with low doses of sulfamethoxazole-trimethoprim is recommended to prevent Pneumocystis jirovecii pneumonia and urinary tract infections. Epstein-Barr virus (EBV)-negative recipients are at high risk of developing cerebral lymphoma if the donor is EBV-positive. In such cases, immunosuppression should be kept as low as possible.

KTRs are at increased risk for developing invasive fungal infections. Administration of nystatin to the duration of admission after transplant may be sufficient for prophylaxis of oral or esophageal candidiasis (90). However, invasive fungal infections typically develop later due to prolonged immunosuppression or delayed immune recovery. In a study of 57,188 KTRs, 1,218 patients developed 1,343 invasive fungal infections, with a median time to infection of 495 days post-transplant. Unspecified mycoses accounted for the largest proportion of infections (37%), followed by aspergillosis (22%). The risk of any fungal infection was higher in patients aged ≥65 years. Diabetes, bacterial pneumonia, and urinary tract infection were identified as the top three clinical risk factors for fungal infection. Each of these categories was further associated with specific individual risk factors (91). Routine antifungal prophylaxis is generally not recommended in KTRs, with the exception of oral prophylaxis against Candida. Current guidelines do not provide specific recommendations for antifungal prophylaxis beyond this indication; therefore, its use should be considered on a case-by-case basis in patients at increased risk. Prophylactic fluconazole at a dose of 200 mg daily for 6–12 months is recommended for all KTRs undergoing transplantation in coccidioidomycosis-endemic areas who do not have evidence of active infection at the time of transplantation (92). Care must be taken with antifungal agents due to potential interactions with immunosuppressive medications (44).

Treatment of invasive fungal infections is challenging. Azoles (ketoconazole, itraconazole, voriconazole, posaconazole) can inhibit P-glycoprotein and CYP3A4, leading to elevated intracellular levels of calcineurin inhibitors. Additionally, amphotericin B carries a nephrotoxicity risk, which can further compound calcineurin inhibitor–related toxicity.

In summary, infection prevention in KTRs is multifaceted. Transplant clinicians should ensure that KTRs adhere to recommended hygiene and lifestyle measures and have received pre-transplant vaccinations against pneumococcus, hepatitis B, COVID-19, and varicella. The use of antibiotics, antifungal, and antiviral agents requires expertise and awareness of potential interactions with immunosuppressive medications. Patient education is as important as medical interventions.

### Tumors

The overall risk of cancer in KTRs is two to three times higher than in the general population of the same age and sex (93).Oncogenic viruses, immunosuppression and altered T cell immunity may contribute to the increased risk of cancer in transplant recipients. Transplant candidates and potential donors should be screened for cancer as part of the assessment process (94). Current recommendations for KTRs are largely based on evidence from the general population. Transplant candidates without a prior history of cancer should be screened for occult malignancies, even if asymptomatic. Conversely, patients with previous malignancy require close surveillance for recurrence or *de novo* cancers. Optimal cancer treatment and immunosuppression management in this setting remain poorly defined. For candidates with prior cancer, a waiting period of 2–5 years after remission—depending on cancer type and stage—is generally recommended. Post-transplant cancer screening should be individualized, considering cancer risk, comorbidities, prognosis, and patient preferences. In KTRs who develop cancer, management includes standard oncologic therapies alongside adjustments to immunosuppression (95). Routine cancer screening, broadly consistent with recommendations for the general population, is advised for kidney transplant recipients; however, no single universally accepted guideline specifically addresses this population. Several professional societies, including those focused on nephrology and transplantation, offer overlapping but sometimes varying recommendations. Therefore, screening strategies should be individualized and combined with appropriate preventive measures, considering patient comorbidities, life expectancy, and overall cancer risk (**Table 3**) (95).

**Table 3:** Proposed overview of preventive strategies and recommended cancer screening protocols, informed by major nephrology and urology guidelines (95).

PREVENTIVE MEASUREs (recommendations)	SUN PROTECTION	Avoid excessive sun exposure and monitor skin changes, including photographing lesions.
	SELF-EXAMINATION	Regularly check your skin and breasts for any suspicious lesions.
	SMOKING	Avoid both active and passive smoking.
	DIET	Follow a diet rich in fruits and vegetables, and low in high-fat foods.
	ALCOHOL	Avoid alcohol or consume it only in moderation.
	WEIGHT MANAGEMENT	Maintain a healthy body weight.
	SEXUAL HEALTH	Practice safe sex to reduce cancer-related infections.
	PHYSICAL ACTIVITY	Engage in regular physical exercise.
SCREENING (method and frequency)	SKIN	Self-examination regularly; annual dermatological evaluation; photograph any new lesion.
	UTERINE CERVIX	Annual Pap test and pelvic examination.
	COLORECTAL	Annual fecal occult blood test; colonoscopy every 10 years.
	PROSTATE	Annual digital rectal exam for men >40 years; ultrasound and biopsy if suspicious. PSA test annually.
	URINARY TRACT	Annual ultrasound of bladder, native kidneys, and graft; annual urine cytology.
	LIVER	Abdomen ultrasound every 6-12 months. Alpha-fetoprotein annually.
	BREAST	Mammography every 1-2 years.
	PTLD	Physical examination every 3 months in the first year, then annually. EBV viremia once in the first week after transplantation, monthly for the first 3–6 months and then every 3 months until the end of the first post-transplant year.

EBV, Epstein-Barr virus; PTLD, post-transplant lymphoproliferative disorder.

Non melanoma skin cancer is by far the most common malignancy among KTRs, particularly in individuals living in sunny regions (96). In addition to the level of immunosuppression, which can facilitate viral-induced carcinogenesis in the skin, the primary—and potentially avoidable—carcinogen responsible for skin cancer is UV radiation. Therefore, in addition to the guidance on sun exposure, sunscreen use, and self-examination of moles provided in the previous paragraph, transplant care providers should perform a baseline skin examination at the time of transplantation and ensure strict dermatologic follow-up assessments.

Kaposi sarcoma (KS) occurs endemically in parts of tropical Africa and certain Mediterranean regions and has been observed among individuals of Jewish and Arab descent. In contrast, it appears sporadically in Europe and North America and remains rare in India. KS is associated with the presence of human herpes virus-8 (HHV8). HHV8 has been regularly found by polymerase chain reaction in all forms of KS (classic KS, AIDS-KS, endemic KS and organ transplant KS), in primary effusion lymphoma, and in Epstein–Barr virus (EBV)-negative post-transplant lymphoproliferative disorders (PTLD) (97). HHV8 genome contains potential oncogenes able to regulate cell proliferation and apoptosis. Therapy for Kaposi’s sarcoma is a reduction of immunosuppression. Chemotherapy, radiation therapy, or surgery is provided for disseminated or recalcitrant disease (98).

PTLD, lung cancer, and renal cancer are among the most frequent malignancies in KTRs. The risk of malignancies is influenced by both the duration and type of immunosuppressive therapy. The risk of developing a lymphoma within the first year after renal transplantation is estimated to be 20 times higher than in the general population (99). Up to 30% of PTLD involves the central nervous system (100), with the most common malignant form being diffuse large B-cell lymphoma, which accounts for only 2% of CNS malignancies in the general population. Many cases of PTLD are associated with prior EBV infection, although cerebrospinal fluid and serum analyses may test negative for EBV DNA, and the specific pathogenic mechanisms remain unclear (101). A definitive diagnosis typically requires a brain biopsy. Careful tapering of immunosuppressive therapy, guided by the patient’s history of graft rejection, is essential. Treatment options include rituximab, chemotherapy, and surgery. More recently, chimeric antigen receptor T cell therapies based on donor leukocyte infusion, virus-specific T cells, T-cell receptor-deficient T cells, lymphoid progenitor cells, and regulatory T cells have been proposed (102).

In summary, cancer prevention in KTRs is a crucial aspect of long-term care. Whenever possible, clinicians should use the lowest effective dose of immunosuppression and may consider mTOR inhibitors (e.g., sirolimus or everolimus) in selected high-risk patients, as evidence indicates a reduced incidence and delayed onset of non-melanoma skin cancers after conversion from calcineurin inhibitors. In contrast, benefits for non-cutaneous malignancies are less evident, and their use must be weighed against higher rates of adverse events and treatment discontinuation (103). Age-appropriate cancer screening (colorectal, breast, cervical, prostate), regular dermatology visits, and monitoring for EBV, HPV, and hepatitis B/C–related malignancies are recommended. Patients should be encouraged to practice sun protection and adopt healthy lifestyle habits, including smoking cessation, moderate alcohol use, a balanced diet, and regular physical activity.

### Neurological complications

A wide spectrum of neurological complications may affect the outcomes of KTRs, potentially resulting in disabling or even life-threatening conditions (104). These complications may stem from the underlying diseases that necessitated transplantation, as well as from dialysis itself (105). These complications may stem from the underlying diseases that necessitated transplantation, as well as from dialysis itself. In addition, immunosuppressive therapies can play a significant role in their development (105). Calcineurin inhibitors can cause tremors and paresthesia; hypertension and diabetes may increase the risk of cerebrovascular accidents; and fungal and viral infections may lead to meningitis and dementia (**Table 4**). Immunosuppressive drugs may exert direct neurotoxic effects. CNIs, particularly tacrolimus, can cause mild symptoms such as tremors and paresthesia (106). Tremors affect roughly one-third of kidney transplant recipients, with severity ranging from mild to severely debilitating (107). In the absence of other causes, tremors usually indicate tacrolimus toxicity, which often occurs independently of drug trough levels. Seizures have been reported in 6-36% of transplant recipients (108), and the most common causes include immunosuppressive drugs, especially tacrolimus. Although high CNIs doses increase seizure risk, seizures may also occur at standard doses, particularly in pediatric patients with metabolic abnormalities (e.g., hyponatremia, hypomagnesemia, hypoglycemia) or infections. Posterior Reversible Encephalopathy Syndrome (PRES) is a serious neurological complication characterized by vasogenic cerebral edema and is closely associated with acute or poorly controlled hypertension. If not promptly recognized and managed, it can lead to permanent deficits (109). KTRs have a higher incidence of cerebrovascular events compared to the general population. This is partly due to pre-existing conditions such as vasculopathy, accelerated atherosclerosis, hypertension, and diabetes—common causes of end-stage renal disease and further worsened by dialysis. Diabetes and age over 40 are significant risk factors for post-transplant stroke (105, 110). Peripheral toxic neuropathy is the most common neurological adverse event in KTRs, with an overall prevalence exceeding 2% (111). Guillain-Barré syndrome (GBS) is the most common form of acute inflammatory polyradiculoneuropathy. Its clinical course typically progresses through three phases: an initial ascending phase characterized by predominantly symmetrical limb weakness and areflexia, reaching its peak within 2–4 weeks; a plateau phase that may persist for several months; and a recovery phase, which can be incomplete in some cases, resulting in residual disability. CMV is the most frequently identified viral trigger of GBS and has been implicated in most cases reported in solid organ transplant recipients. When GBS is suspected in KTRs, especially in the context of a possible viral etiology, the optimal treatment approach appears to be a combination of intravenous immunoglobulin or plasmapheresis along with antiviral agents. This combined strategy has been associated with better outcomes and should be considered in all cases where a viral cause cannot be ruled out (112). Fungal infections are less frequent, but they are burdened by a high mortality rate, with cryptococcus neoformans displaying the highest incidence in this category (113).

**Table 4:** Neurological complications in kidney transplant recipients—Etiologies, risk factors, and clinical manifestations (102).

Neurological conditions	Etiologies and risk factors
Immunosuppressive Medications Associated Symptoms	Calcineurin inhibitors may cause mild symptoms such as tremors or paresthesia, or severe effects like disabling pain and leukoencephalopathy.
Stroke	Risk is increased by hypertension, diabetes, and accelerated atherosclerosis.
Peripheral Neuropathy	Mononeuritis or polyneuritis may occur. Guillain-Barré syndrome can be triggered by Cytomegalovirus or Campylobacter jejuni infection.
Infection	Acute meningitis is commonly caused by Listeria monocytogenes. Subacute or chronic meningitis by Cryptococcus neoformans. Focal brain infections may result from Aspergillus fumigatus, Toxoplasma gondii, or Nocardia asteroides. Dementia may be due to polyomavirus JC or other neurotropic viruses.
Tumor	Lymphomas are the most frequent brain tumors, typically associated with Epstein–Barr virus infection and/or profound immunosuppression.

## Conclusions

Active patient engagement is essential for preventing post-transplant complications. Education should be prioritized, with a strong emphasis on preventive strategies. Beyond guidance on immunosuppression, patients must receive clear, practical instructions to minimize avoidable risks and enhance long-term graft survival. Many serious complications—including infections, cardiovascular disease, and cancer—are largely preventable through comprehensive education, close monitoring, and timely intervention. The clinician’s role extends beyond assessing kidney function to ensuring prompt recognition and management of all complications, even minor ones. Clinical judgment should take precedence over reliance on biomarkers, which may not always provide accurate or timely information. In conclusion, sustained vigilance—grounded in clinical judgment, comprehensive patient education, and timely intervention -are essential to preserving graft function and ensuring long-term patient survival.
